# Benchmarking total knee replacement constructs using noninferiority analysis: the New Zealand joint registry study

**DOI:** 10.1186/s12891-021-04606-w

**Published:** 2021-08-23

**Authors:** MC Wyatt, CF Frampton, MR Whitehouse, KC Deere, A Sayers, D Kieser

**Affiliations:** 1 Massey University, Palmerston North, New Zealand; 2 University of Otago, Dunedin, New Zealand; 3 University of Bristol, Bristol, UK

**Keywords:** Total knee replacement, Noninferiority analysis, Benchmarking

## Abstract

**Background:**

The aim of this study was to compare the relative performance of total knee replacement constructs and discern if there is variability in performance in currently commonly used prostheses in the New Zealand Joint Registry (NZJR) using a noninferiority analysis.

**Methods:**

All patients who underwent a primary total knee replacement (TKR) registered in the NZJR between 1st January 1999 to June 2020 were identified. Using a noninferiority analysis, the performance of total knee replacement prostheses were compared with the best performing contemporary construct. Construct all-cause revision rate was estimated using the 1-Kaplan Meier survival function method to estimate net failure. The difference in all-cause revision rates between the contemporary benchmark and all other constructs was tested.

**Results:**

In total 110 183 TKR were recorded and 25 constructs (102 717 procedures) had > 500 procedures at risk at 3 years post-primary of which 5 were inferior by at least 20 % relative risk of which, one was inferior by at least 100 % relative risk. 14 constructs were identified with > 500 procedures at risk at 10 years with 5 inferior by at least 20 %, of which 2 were inferior by > 100 % relative risk.

**Conclusions:**

We discerned that there is great variability in construct performance and at all time points, greater than 25 % of constructs are inferior to the best performing construct by at least 20 %. These results can help inform patients, clinicians and health care funders when considering TKR surgery.

## Introduction

When patients, surgeons or commissioners are considering which total knee replacement (TKR) to use it is understandable that many assume that the different constructs function equally. However in a recent study of the largest arthroplasty database in the world, there was in fact wide variation in total knee replacement construct performance [1]. The extent to which this is transparent to patients, clinicians and health funders is unknown. This lack of transparency is profoundly important with respects to medical device safety as recently highlighted in the Cumberlege review [2].

Greater than 8000 total knee replacements are performed annually in New Zealand and there are a large number of implants from which to choose (nzoa.org.nz). The National Joint Registry of New Zealand (NZJR) was established to monitor the performance of joint replacement implants and identify poorly performing implants. It has not yet reported on identifying exceptionally-performing implants or comparing implants to the best performing at specific time points. The NZJR publishes the unadjusted failure rates expressed as a prosthesis time incidence rate (PTIR) of the TKR brand combinations used in its annual reports. In New Zealand the Pharmaceutical Management Agency (Pharmac) is the agency that decides which TKR products are subsidised for use in public hospitals [3]. Understanding which devices should receive subsidies is important to ensure joint replacement is as cost effective as possible. In the private sector the decision of which TKR implants are used is at the surgeon’s discretion.

Promoting perceived good practice has been done by other organizations such as the Orthopaedic Device Evaluation Panel (ODEP) in the UK [4] and in Australia, the Australian superior clinical performance programme [5] but these commonly use an perhaps arbitrarily-defined static external benchmark. ODEP for example recommends an external benchmark of all-cause revision rate < 5 % at 10 years [4].

Adequately-powered randomised controlled trials comparing implants provide the best scientific evidence by minimizing bias and confounding through randomization. However such trials are expensive both financially and in terms of time investment. Without randomized controlled trial evidence, prospective national registers of joint replacement (national joint registries) are arguably the best current sources of evidence of prosthesis performance. Like all observational data it has inherent limitations that make the interpretation of the outcomes of prosthesis or prosthesis constructs challenging. For example cause/effect relationships cannot be deduced rather analyses can determine strengths of associations. Furthermore there is the potential for confounding factors, biases and chance to influence the apparent results.

The PTIR’s reported by the NZJR gives an indication of performance in absolute terms but not a head-to head comparison. Sayers et al. proposed an analysis paradigm which used a noninferiority design against an external benchmark [6]. In this simulation study the 1-Kaplan-Meier methodology gave an unbiased estimate of “net failure” with or without the addition of a competing risk. However the use of an internal rather than external benchmark can be justified as this is dynamic and responds to changes in all-cause revision rate outcomes over time. In a noninferiority trial with all-cause revision rate as the primary outcome, a comparator and internal reference can be compared to ensure that the comparator treatment is within a clinically acceptable range (noninferiority margin) of performance at a specified time point [7, 8]. The choice of the appropriate commonly-used reference to define the best practice benchmark was the construct, used in sufficient numbers to maximally protect against chance, clustering and surgical performance variation, with the lowest all-cause revision rate in the registry. Construct is influenced by both age and gender [9] therefore the choice of reference should ideally reflect this specificity. There has recently been shown great variability demonstrated in TKR construct performance in the UK at 3, 5, 7 and 10 year time points using an internal benchmark [1]. We note that the Australian Joint Registry has used a noninferiority benchmarking approach to report since 2018 and more widespread standardised Registry reporting in this manner may add clarity. Whether similar findings occur in New Zealand is unknown.

The aim of this study therefore was to compare the performance of TKR prosthesis constructs compared to the best-performing construct, the benchmark, using a noninferiority analysis and illustrate any variability in performance using the NZJR. Constructs were examined against noninferiority margins of 20 and 100 % relative risk (i.e. double the revision rate) at 3, 5, 7 and 10 years following surgery.

## Methods

### Patients and data sources

The NZJR was established in 1998 and has a > 96 % data capture rate of all joint replacement surgeries [10]. Prospective entry of data into the NZJR is a mandatory requirement of all members of the New Zealand Orthopaedic Association with all data secured in Christchurch, New Zealand and all patients provide written consent for their data to be included. One of the authors (CF) accessed the database to acquire data specifically for this study. Deidentified data of all patients undergoing primary TKR from the NZJR inception to 1st June 2020 was available for analysis and the NZJR is linked directly with the NZ database for births and deaths.

Brands of TKR constructs were subdivided by fixation (cemented or uncemented), mobility of the bearing (mobile or fixed) and degree of constraint (cruciate retaining CR, posterior stabilized PS). NZJR and NJR data have shown that these characteristics influence revision rates and were therefore treated each subdivision as a separate construct.

No formal Institutional Review Board (IRB) approval was required as this was a review of the NZJR which already has IRB approval for publication of results stored in its registry.

### Primary exposure

The primary exposure used in this analysis was the TKR prosthesis construct. Construct groupings were defined using data recorded by the NZJR and based on the catalogue numbers of individual TKR prosthesis.

### Statistical methods

Prosthesis construct all-cause revision was estimated using the 1-Kaplan-Meier method, that is, an estimate of “net failure ^**“**^[11]. All-cause revision was defined using the first linked surgical revision; patients were censored at death. A revision was defined as a new operation in a previous TKR during which one or more of the components was exchanged, removed, manipulated or added. It included excision arthroplasty and amputation, but not soft tissue procedures.

The reference construct was the construct with the lowest all-cause revision rate with at least 1 000 patients at risk at the time point of interest for all procedures and for each stratum. The choice of 1 000 procedures of the same construct was based on simulation work by Sayers et al. [6] which demonstrated that 1 000 procedures at risk will give rise to a CI width of ~ 3 % (± 1·5 %). The difference in stratum-specific failure probabilities compared with the reference were calculated at 3, 5, 7 and 10 years for all prosthesis combinations that had 500 or more patients still at risk and also then stratified by gender. The difference and 95 % CI of the difference between the comparator prosthesis construct and the reference prosthesis construct was estimated at the specified time points. The standard error (SE) of the difference was constructed using a pooled estimate of the Greenwood [12]. A Wald test was then used to compare the difference between the reference and the test prosthesis.

The noninferiority margins chosen to illustrate the sensitivity of the choice were 20 and 100 % relative risk. The former represents the typical threshold used in clinical trials and the latter represents a doubling in cumulative probability of failure, as this is an easily interpretable outcome. Results are graphically reported for all comparator prosthesis constructs meeting the criterion at each time point of interest. These figures show the failure difference for each construct compared to the reference and the number of constructs still at risk. The threshold for graphical presentation, 500 procedures at risk, was chosen based on the previous work of Sayers et al. [6] as this would give rise to an individual CI width of ~ 5 % (± 2·5 %), implemented by Deere at al. [1] and because it complements the number of procedures at risk used by ODEP when evaluating devices at 10 years (www.odep.org.uk).

**Fig. 1:**
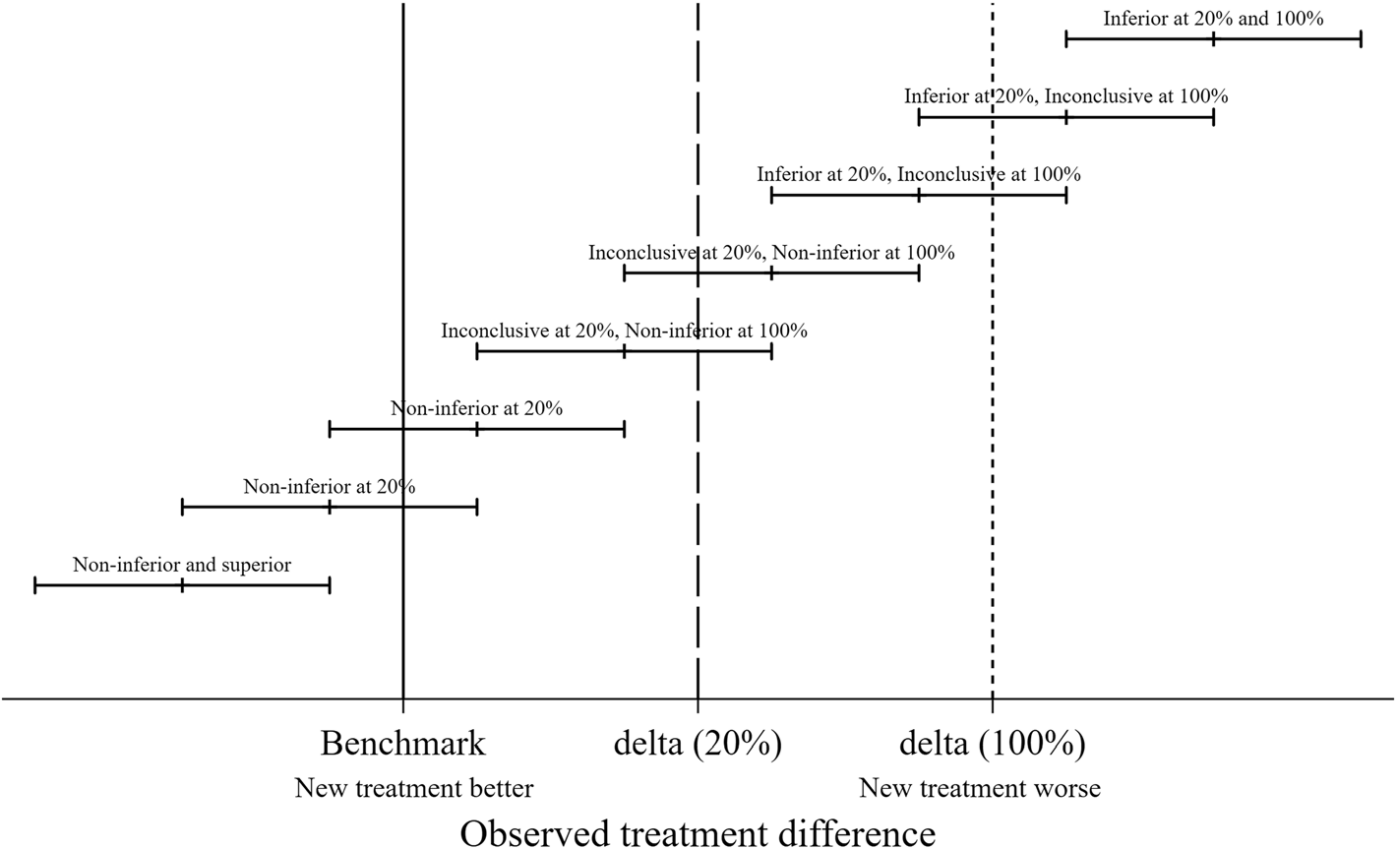
Schematic representation of inferiority and noninferiority

**Fig. 2:**
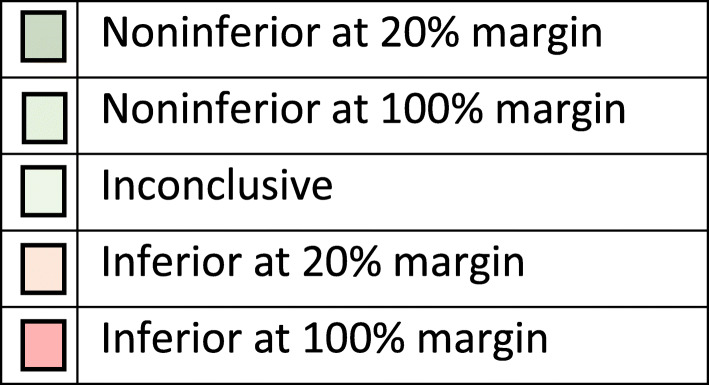
Key for colour coding in Figs. 3-6. If no constructs were in a particular band the colour was not displayed

Prosthesis constructs were either classified as noninferior, inconclusive or inferior by comparison with the two noninferiority margins and the classification shown in the five resultant groups. If the lower CI limit is above the 100 % noninferiority margin, the prosthesis construct was classified as inferior at the 100 % margin. If the lower CI limit was above the 20 % noninferiority margin but not above the 100 % inferiority margin the construct was classified as inferior at the 20 % margin. If the upper CI limit was below the 100 % inferiority margin, the construct was defined as noninferior at the 100 % margin. If the upper CI limit was below the 20 % inferiority margin, the construct was defined as noninferior at the 20 % margin. All the other results were determined to be inconclusive in terms of both 20 and 100 % margins for noninferiority. Figure 1 provides a graphical representation of the rationale for the classification for a single noninferiority margin and Fig. 2a key for subsequent figures.

## Results

There were 110,183 primary TKR included in the NZJR from inception to 1st June 2020 from which 25 constructs (102 717 procedures) were available with > 500 remaining at risk for the 3 year analysis. A detailed description of noninferiority across all procedures is provided.

### Noninferiority: all procedures

**Fig. 3:**
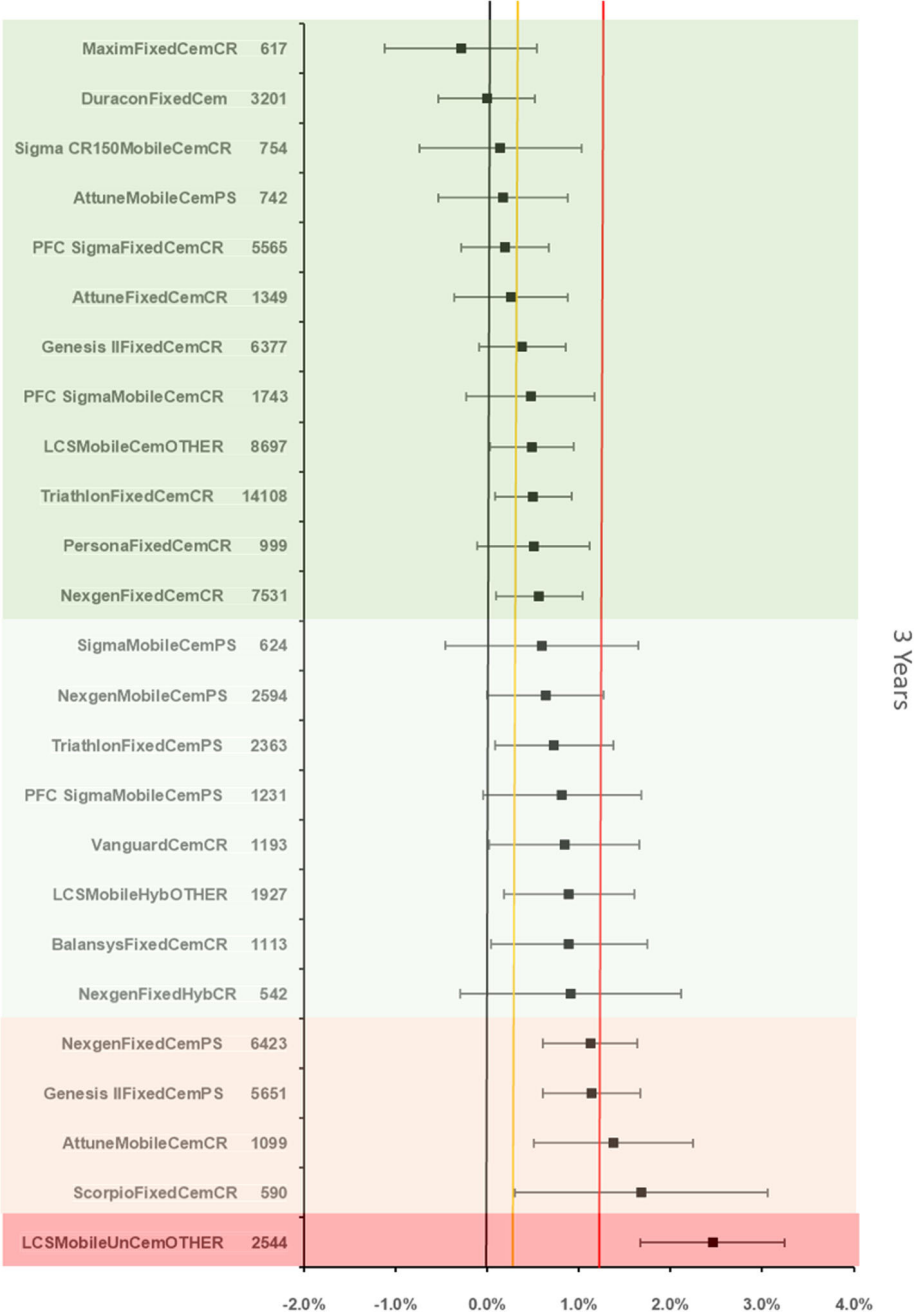
Difference in all-cause revision rates of implanted constructs compared with a contemporary reference (DuraconFixedCem (1.22 %, 95 % CI 0.85–1.6) at 3 years

The references prosthesis construct at 3 years was identified as the Duracon Fixed bearing cemented TKR. There were 3201 remaining at risk and the all-cause revision rate was 1.22 % (95 %CI 0.85–1.6). There were 24 other constructs with > 500 at risk. 4 were inferior by at least 20 % relative risk of which 1 construct was identified as inferior to the reference by > 100 % relative risk (Fig. 3).

**Fig. 4:**
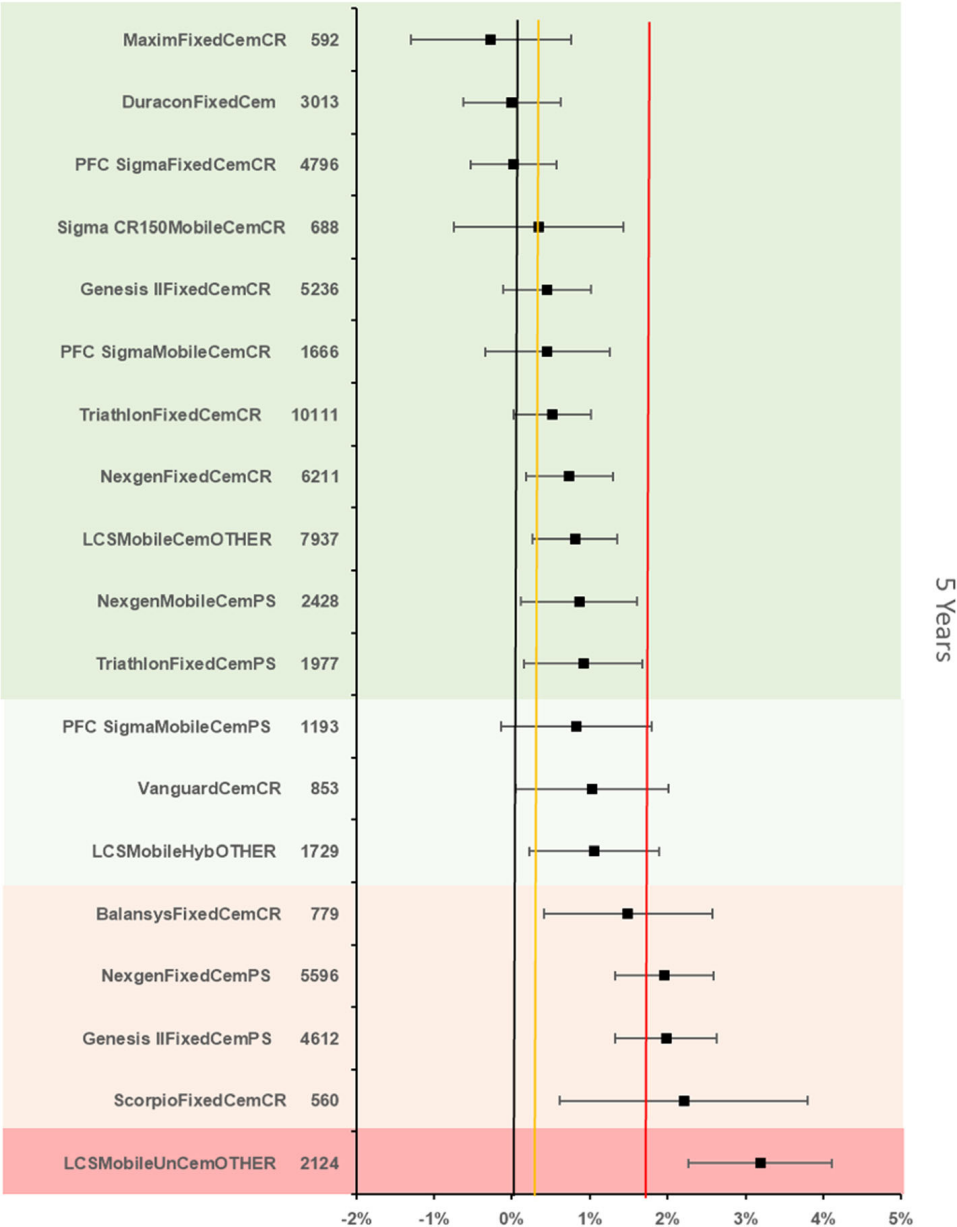
Difference in all-cause revision rates of implanted constructs compared with a contemporary reference (DuraconFixedCem (1.7 %, 95 % CI 1.26–2.14) at 5 years

The references prosthesis construct at 5 years was again identified as the Duracon Fixed bearing cemented TKR. There were 3013 remaining at risk and the all-cause revision rate was 1.7 % (95 % CI 1.26–2.14). There were 18 other constructs with > 500 at risk. 4 constructs were inferior by at least 20 % relative risk of which 1 construct was inferior to the reference by > 100 % relative risk (Fig. 4).

**Fig. 5:**
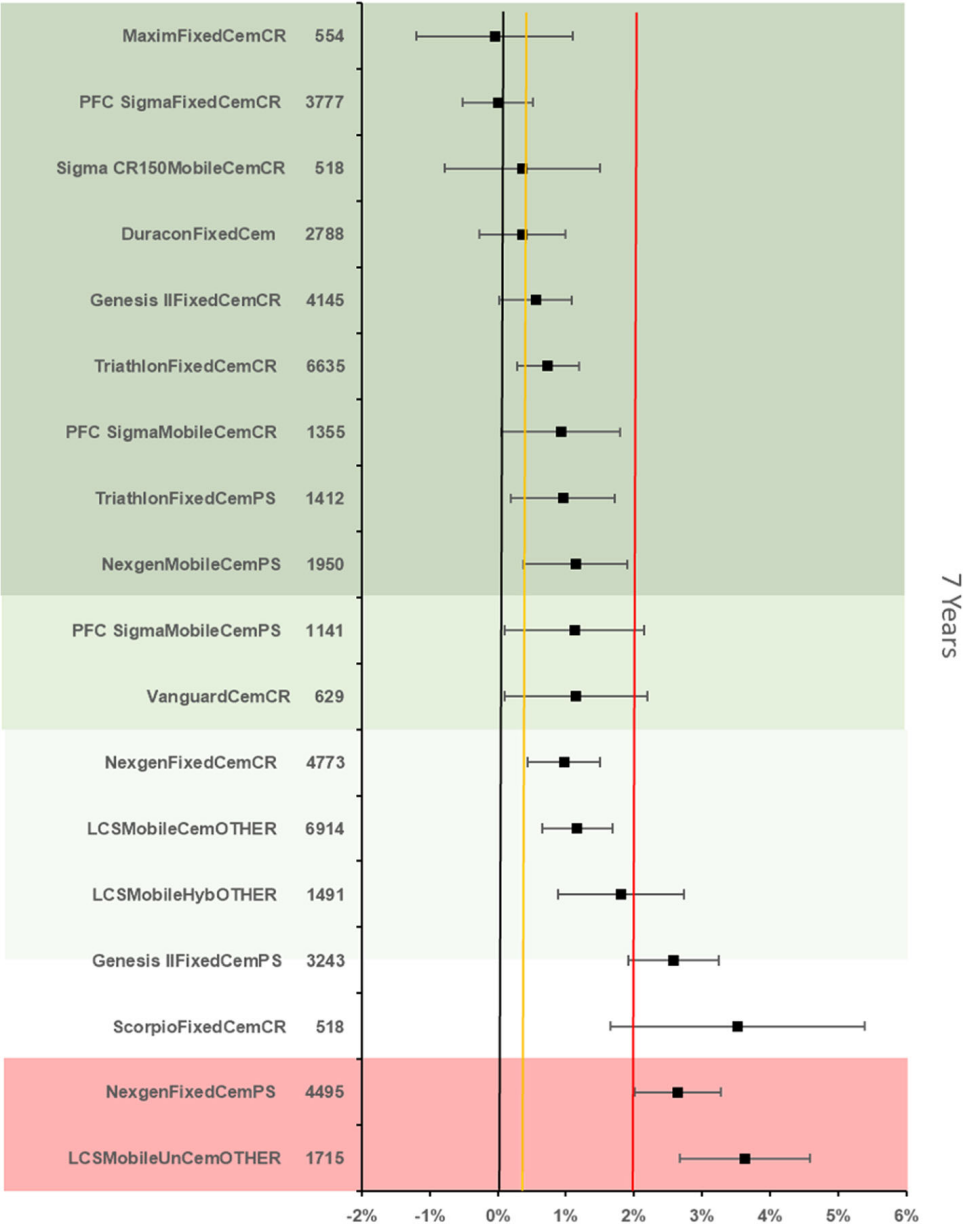
Difference in all-cause revision rates of implanted constructs compared with a contemporary reference (PFC SigmaFixedCemCR (1.98 %, 95 % CI 1.62–2.34) at 7 years

The references prosthesis construct at 7 years was identified as the PFC Sigma Fixed bearing cemented cruciate retaining construct. There were 3777 remaining at risk and the all-cause revision rate was 1.98 % (95 % CI 1.62–2.34). There were 16 other constructs with > 500 at risk. 7 constructs were identified as inferior to the reference by > 20 % relative risk of which 2 were inferior by > 100 % relative risk. (Fig. 5).

**Fig. 6:**
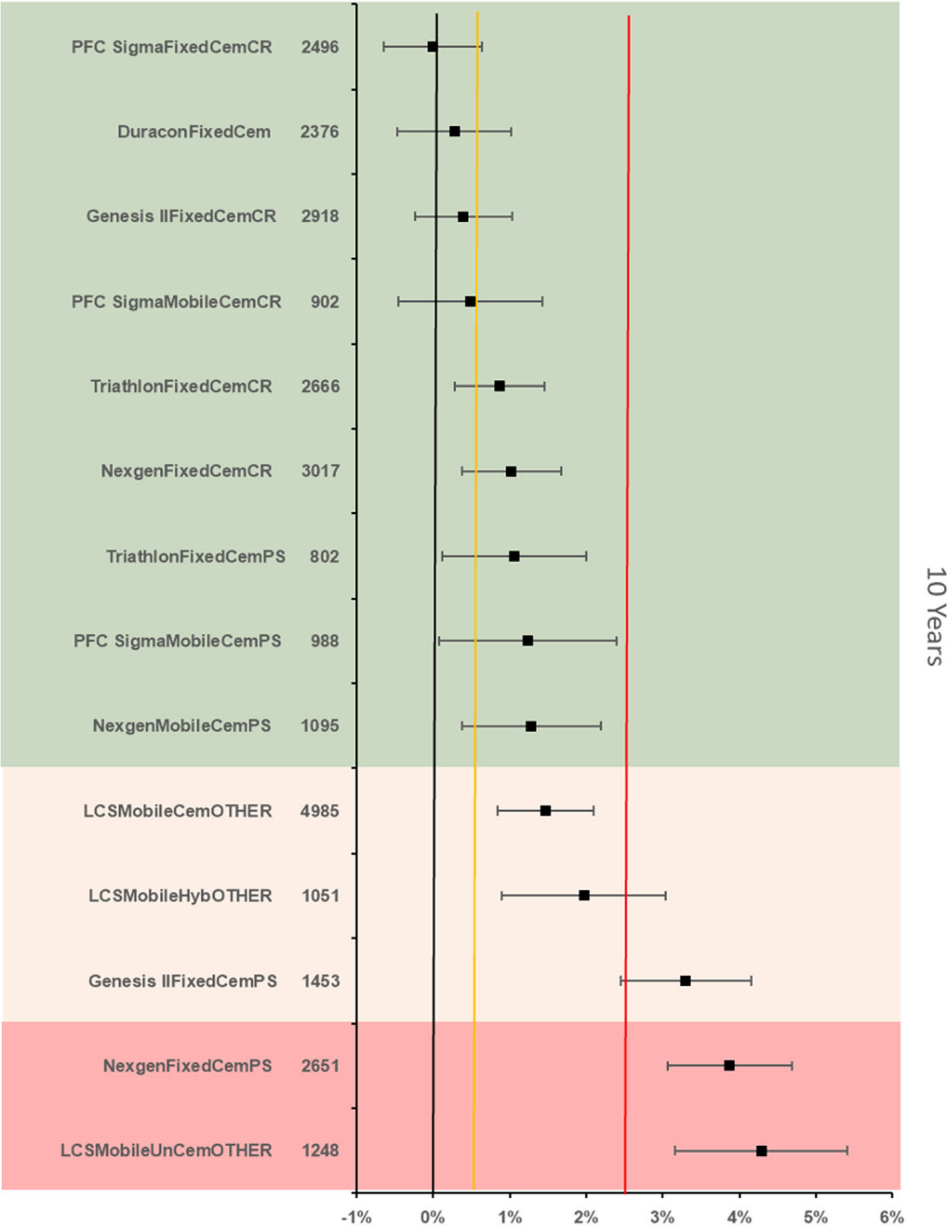
Difference in all-cause revision rates of implanted constructs compared with a contemporary reference (PFC SigmaFixedCemCR (2.58 %, 95 % CI 2.13–3.04) at 10 years

The references prosthesis construct at 10 years was identified as the PFC Sigma Fixed bearing cemented cruciate retaining construct. There were 2 496 remaining at risk and the all-cause revision rate was 2.58 % (95 % CI 2.13–3.04). There were 13 other constructs with > 500 at risk. Five constructs were identified as inferior to the reference by > 20 % relative risk of which 2 constructs were inferior by > 100 % relative risk (Fig. 6).

## Discussion

We have shown in 102 717 primary TKRs the relative performance of implanted constructs in comparison to an internally selected reference. There is substantial variation in the performance of TKR prosthesis constructs. A noninferiority approach to best practice benchmarking provides an immediate comparison of commonly used implanted prosthesis constructs compared with an internal commonly-used reference and conveys distinct advantages as opposed to component/years in the NZJR annual reports, standard Kaplan-Meier analyses in the NZJR reports, or categorical grades provided by organisations such as ODEP.

Our study also shows that at all time points the best practice benchmarks were cemented fixed-bearing cruciate-retaining constructs. Furthermore the reference construct at 3 and 5 year time point is no longer available in New Zealand. The benchmark prosthesis at 7 and 10 years was the PFC Sigma cemented Fixed bearing CR and this implant is soon to be superseded. One could argue therefore that these findings therefore challenge the rationale for the introduction of the newer “improved” constructs. In addition choosing “the benchmarks” may be also challenging in this context.

Notwithstanding the above the two most commonly implanted TKR in NZ today are the Triathlon cemented fixed bearing CR and the Attune cemented fixed bearing CR. In the overall comparisons these two currently most-commonly implanted constructs were both non-inferior to benchmark at 3 years. The Triathlon, Genesis II and Nexgen cemented fixed bearing CR constructs were all non-inferior to benchmark at 3, 5, 7 and 10 years. This strongly suggests that these currently-available combinations could appropriately be used for the majority of patients. This is particularly relevant for inexperienced surgical teams, as they can focus training on, and become expert with, a single prosthesis construct. Potentially this may reduce the risk of technical error, to be cost saving through bulk purchasing arrangements and via a reduction in failure rates. However the availability of PS TKR’s is necessary in certain situations such as significant valgus deformity, post-patellectomy and in the context of inflammatory arthritis. Similarly a surgeons approach to patellar resurfacing in TKR may be always never or selectively which adds another layer of complexity to interpreting the results.

The absolute all-cause revision rate of commonly-used constructs is relatively low, and < 5 % in many instances. Interestingly uncemented constructs performed less well than cemented compared constructs in accordance with a recent study [13]. In the study of National Joint Registry of England, Wales, Northern Ireland and the Isle of Man Deere et al., applying the same methodology, found that at 3 and 5 years the benchmark was a cemented CR fixed bearing TKR which is consistent with our study. Interestingly at 7 years an uncemented CR TKR was benchmark. At 10 years there was the same benchmark TKR as in our study of the NZJR. This similarity of findings suggests that the results are generalizable.

This analysis has a number of important strengths. Firstly, the presentation of data allows surgeons, patients and policy makers to directly compare commonly-used prosthesis constructs to an internal reference construct. Secondly the constant application of benchmarking methodology and similar observed trends across both the New Zealand and English and Welsh National Joint Replacement Registers suggests the results are generalizable and will be useful to both patients, surgeons, and policy makers.

Our study has a number of limitations; case-mix adjustment by stratification is difficult to assimilate and despite efforts to restrict confounding factors, residual and unmeasured confounding factors are likely to be present. Whilst a priori and as recommended by ISAR we intended to perform subgroup analysis for both age and gender there were insufficient numbers to stratify the risk in this way. We have also not compared constructs to individual components. The ability to interpret analyses from a causal perspective is limited when using observational data. It is also known that revision rate is influenced by factors such as the primary indication and the severity of preoperative knee disease. In addition whilst all-cause revision as an endpoint is recommended by ISAR we acknowledge that a revision may not be carried out because of the implant failing per se. Moreover prosthetic joint infection has been shown in the NZJR to be the most common cause of revision for TKR [14]. However whilst we have assumed a constant risk of PJI requiring revision across all constructs we do not feel that this skews our results as the incidence of PJI requiring revision is low.

We have applied the methodology of Deere et al. to the NZJR. However we acknowledge that this approach varies from some of the recommendations of ISAR (https://www.isarhome.org/home) in that we combined the results of both CR and PS TKR constructs, did not perform an additional superiority analysis and set benchmarking criteria of 1000 at risk and comparators at 500 at risk at each time point.

The results from this study have potential implications for the way both practicing surgeons, purchasers and patients approach total knee replacement. With this presentation of data it is now possible for all parties to have a considered approach to either purchasing a knee replacement or deciding to have surgery with a particular construct and the likely chances of experiencing a revision. With specific reference to New Zealand policy of subsidization of prosthesis constructs used in public hospitals, it is essential that purchasers have access to all local and globally relevant and independent sources of data to ensure public money is used in the most cost-effective manner possible, ensuring as many patients will be treated as possible within the available budget constraints.

For new surgeons, or surgeons looking to optimize the care of their patients, they now have an independent and detailed source of data which compares a wide variety of prosthesis constructs using clinically relevant strata. This will ensure they can pick prostheses that match their surgical competencies or reflect on their need to seek further training, for example in the use of particular prostheses, to ensure they can use implants with a strong track record of performance. Lastly, we hope detailed data will be made available to patients in order to facilitate the shared decision-making process required to inform patients of the risk of revision before deciding to undergo surgery.

## Data Availability

The datasets generated and/or analysed during the current study are available in the New Zealand Joint Register (nzoa.org.nz).
